# What Is the Evidence for Early Mobilisation in Elective Spine Surgery? A Narrative Review

**DOI:** 10.3390/healthcare7030092

**Published:** 2019-07-18

**Authors:** Louise C. Burgess, Thomas W. Wainwright

**Affiliations:** 1 Orthopaedic Research Institute, Bournemouth University, Bournemouth BH8 8EB, UK; 2 Physiotherapy Department, The Royal Bournemouth and Christchurch Hospitals NHS Foundation Trust, Bournemouth BH7 7DW, UK

**Keywords:** spine surgery, early mobilisation, enhanced recovery after surgery (ERAS), physiotherapy, rehabilitation

## Abstract

Early mobilisation is a cornerstone of Enhanced Recovery after Surgery (ERAS) and is encouraged following spinal procedures. However, evidence of its implementation is limited and there are no formal guidelines on optimal prescription. This narrative review aimed to evaluate the evidence for the effect of early mobilisation following elective spinal surgery on length of stay, postoperative complications, performance-based function and patient-reported outcomes. Four trials (five articles) that compared a specific protocol of early in-hospital mobilisation to no structured mobilisation or bed rest were selected for inclusion. Nine studies that investigated the implementation of a multimodal intervention that was inclusive of an early mobilisation protocol were also included. Results suggest that goal-directed early mobilisation, delivered using an evidence-based algorithm with a clear, procedure-specific inclusion and exclusion criteria, may reduce length of stay and complication rate. In addition, there is evidence to suggest improved performance-based and patient-reported outcomes when compared to bed rest following elective spinal surgery. Whilst this review reveals a lack of evidence to determine the exact details of which early mobilisation protocols are most effective, mobilisation on the day of surgery and ambulation from the first postoperative day is possible and should be the goal. Future work should aim to establish consensus-based, best practice guidelines on the optimal type and timing of mobilisation, and how this should be modified for different spinal procedures.

## 1. Introduction

Over the last twenty years, the significant growth and adoption of Enhanced Recovery after Surgery (ERAS) or “fast-track” pathways represent a paradigm shift in how surgical care is delivered for a number of procedures [1]. The multimodal, multidisciplinary approach to the care of a patient comprises a combination of evidence-based interventions in the perioperative period that aim to reduce convalescence by reducing the stress of the operation to retain anabolic homeostasis [1]. This involves preparing the patient for surgery through assessment and education, ensuring they have the best possible management during their operation (including minimally invasive surgery, pain control and optimal fluid management) and expediting their postoperative recovery (for example, through early feeding and mobilisation) [2,3,4].

Early mobilisation is considered a cornerstone in enhanced recovery pathways, and is strongly recommended as part of ERAS guidelines in many surgical disciplines [5,6,7]. The traditional practice of bed rest following a surgical procedure has been associated with negative outcomes [8], such as a greater risk of thromboembolism, pneumonia, muscle wasting and physical deconditioning [9]. Early mobilisation is advocated to reduce respiratory complications [10,11] and thromboembolic events [12,13] and can improve functional capacity following major elective surgery [14,15]. Despite the recognised importance of early mobilisation in ERAS pathways, compliance to mobilisation targets has been reported as low, and those who fail to mobilise early post-surgery are reported to have a higher risk of complication [16].

ERAS has recently been applied to spinal surgery [17,18,19,20,21,22,23,24], and has been advocated for accelerating postoperative recovery and improving longer term outcomes [25]. A recent systematic review has summarised the implementation of ERAS to spine surgery, with results demonstrating a reduced length of stay with no increase in rates of readmission or complication [26]. Early mobilisation is encouraged following spinal procedures and has been linked to reduced morbidity (respiratory decompensation, urinary tract infections, deep vein thrombosis/pulmonary embolism, sepsis or infection) along with reduced average length of stay. However, the evidence summarising its effectiveness has so far been limited [27]. Encouraging patients to mobilise soon after surgery makes physiological sense. However, several questions regarding the clinical implementations of this intervention following spine surgery remain unanswered. For example, what does “early mobilisation” mean for spinal surgery patients? Does early mobilisation have an effect on short-term recovery goals?

Whilst it is generally agreed that patients should get out of bed and ambulate, if they can, as soon as possible, there are currently no formal recommendations on the optimal time to initiate mobilisation in the postoperative phase for spinal patients [28]. Consensus-based best practice guidelines for postoperative care following posterior spinal fusion for adolescent idiopathic scoliosis recommend sitting on the edge of the bed, ambulation and physical therapy twice daily from postoperative day one [29]. However, to date, there are no mobilisation guidelines for adults undergoing elective spinal surgery. High levels of fear of re-injury through movement and exercise (kinesiophobia) have been reported for this patient group [30] and given that increased levels of kinesiophobia have been associated with decreased postoperative activity [31] and adoption of fear-avoidance behaviour [32], the importance of safely developing mobilisation protocols is clear. We therefore aim to review the current evidence for early mobilisation after elective spinal surgery for adults in order to continue the development of enhanced recovery pathways for these procedures.

## 2. Materials and Methods

A web-based literature search was completed in April 2019 and the databases sourced included Cochrane Library, CINAHL (The Culmative Index to Nursing and Allied Health Literature) Complete, Medline Complete and PubMed, accessed through Bournemouth University’s online library. The search aimed to yield studies to assess the effect of early mobilisation (also termed early ambulation) on outcomes from spinal surgery. The research question has been described using the PICOS (Population, Intervention, Comparison, Outcome measures, Study design) criteria in Table 1.

A search strategy (Box 1) was developed to capture articles that had evaluated the effects of early mobilisation on outcomes from spinal surgery. The search reviewed titles and abstracts of the available, peer-reviewed literature published from the earliest record on file until April 2019. Randomised and non-randomised clinical trials and retrospective analyses were included, given the paucity of evidence in the area of early mobilisation following spine surgery. Secondary searching was also conducted, whereby the reference lists of included studies were scanned for relevant citations. Finally, a special edition of Neurosurgical Focus was released in April 2019 [33], which included 15 new articles on the principles of ERAS and how they relate to spine surgery. These articles and their reference lists were also assessed for eligibility.

Studies were included if they were: (1) conducted in cohorts of adults (over 18 years old) receiving elective surgery, or revision surgery, on the lumbar, cervical, thoracic or sacrum region of the spine; (2) a specific protocol of early, in-hospital mobilisation (with out of bed activities starting no later than postoperative day one); and (3) compared to a control group receiving no structured mobilisation, bed rest or pre-intervention implementation data (pre/post analysis).

Box 1 Search Strategy. * indicates truncation; ERAS—enhanced recovery after surgery.

“Spin* surgery” OR “spine surgery” OR “spinal surgery” OR “back surgery” OR “spin* fusion” OR “spin* stenosis” OR spondylodesis OR “spin* disease” OR “disc surgery” OR “lumbar surgery” OR scoliosis OR cervical OR kyphosis OR osteomy AND “early mobilisation” OR “early ambulation” OR ambulat* OR “early mobilization” OR “early mobility” OR “enhanc* recover** OR “fast-track” OR “fast-track” OR “ERAS” OR “rapid surgery” OR “rapid-surgery” OR “rapid-recovery” OR “rapid recovery” OR “multimodal pain” NOT “spinal cord injury” NOT (fracture or injury)

Studies were excluded if: (1) they were conducted in cohorts of patients undergoing non-elective spinal surgery, surgery to the coccyx region of the spine or surgery for spinal cord injury; (2) conducted in children (under 18 years old); (3) the mobilisation protocol was not described or started later than postoperative day one; (4) no outcomes of interest were measured; (5) there was no comparison patient group (either a control group or pre/post intervention data); (6) there was no access to the full text article; or (7) the article was not available in the English language.

Given that this is a narrative review, studies were included in a separate analysis if the early mobilisation had been delivered as part of a multimodal or “enhanced recovery” intervention, although it was acknowledged that the results of the study could not be solely attributable to the early mobilisation intervention. For this reason, no methodological assessment was completed on these studies. The predetermined inclusion/exclusion criteria are listed in Table 1, with study specific characteristics defined using the PICOS criteria.

### 2.1. Outcome Measures

The outcome measures of interest for this review included postoperative complication rates, length of stay in hospital, readmission rate, performance-based functional outcomes, patient-reported outcome measures and patient disposition. Data were also extracted on the mobilisation protocol and adherence to intervention.

### 2.2. Data Extraction

Once studies had been identified, duplicates were removed and then the titles and abstracts of the remaining articles were screened for the inclusion and exclusion criteria by two independent researchers (L.C.B. and T.W.W.). Once clearly ineligible articles had been removed, full-text screening was conducted by two researchers (L.C.B. and T.W.W.). Any discrepancies between researchers were resolved through discussion with the research team. Data were then extracted from the articles by two independent researchers (L.C.B. and T.W.W.) into a Microsoft Excel spreadsheet. Data were collected on outcome measures, study design, patients, and early mobilisation protocol.

### 2.3. Data Analysis

Data were analysed using a narrative synthesis approach. Where available, differences in outcome measure means were the primary summary measure.

### 2.4. Quality Assessment

A modified Downs and Black checklist [34] was used to assess the risk of bias within the studies yielded in this review. The 27-item checklist was chosen as it is suitable for the appraisal of both randomised and non-randomised clinical trials, and has been shown to have good intra-rater and inter-rater reliability [34]. It has previously been used in systematic reviews with various study designs, and has also been amended to suit the structure of the review in which it was utilised [35,36,37]. Therefore, a modified version (items: 5, 9, 14–16 and 23–27 removed) of the Downs and Black checklist was utilised. The adapted version consisted of 17 items (items 1–4, 6–8, 10–13 and 17–22 (Appendix A)) from the original list, with a maximum possible score of 17 (the higher scores indicate superior quality). The first ten items on the scale relate to reporting and include aims, outcome measures and results. Item 11–13 relate to external validity and consider whether results from the study can be generalised to a wider population. Items 17–22 relate to internal validity (bias and selection bias). Risk of bias was assessed by two independent assessors (L.C.B. and T.W.W.) and any discrepancies were resolved through discussion.

## 3. Results

**Figure 1:**
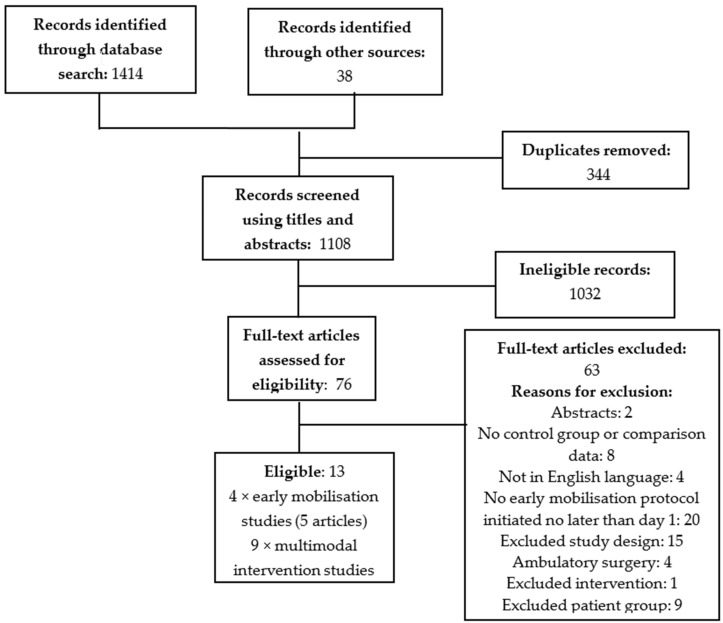
Study identification flow chart (adapted from the Preferred Reporting Items for Systematic Reviews and Meta-Analysis flowchart) [49].

The search generated 1414 results and an additional 38 articles were sourced through secondary searching. Once duplicates (344) were removed, the titles and abstracts of the remaining 1108 results were screened for eligibility. One thousand and thirty-two irrelevant articles were removed, and 76 studies underwent full-text screening. A further 63 articles were removed (abstracts (2); no control group or comparison data (8); not available in the English language (4); early mobilisation protocol not described or initiated later than postoperative day one (20); excluded study design (15); ambulatory surgery (4); excluded patient group (9); or neural mobilisation intervention (1). Four studies (five articles) that compared the effects of early mobilisation to no mobilisation [28,38,39,40,41] (Table 2), and nine studies that compared the implementation of a multimodal intervention that was inclusive of an early mobilisation protocol [21,24,42,43,44,45,46,47,48] (Table 3) were included in the results synthesis. The flowchart of the study selection process is presented in Figure 1.

### 3.1. Characteristics of Included Studies

One randomised controlled trial [39], one ambispective study [38], one retrospective analysis [28] and one pilot randomised controlled trial [41] were yielded. The characteristics of these studies are summarised in Table 2 and their quality assessment scores in Appendix A. The methodological quality of the studies on a whole ranged from 88–94% (mean score: 90%). Two of the four studies were deducted points for item 19 (compliance) as adherence to exercise intervention was reported to be at 85% [39] and 87.3% per month [28]. One study was deducted a point from item 22 as their study participants were not recruited over the same period of time [28] and as the study by Qvarfordh et al. [41] was a pilot study and did not complete a statistical analysis, points were deducted from items ten (probability values) and 18 (statistical tests).

### 3.2. Sample Characteristics

There was a total of 922 participants included in the four studies that compared an early mobilisation to no mobilisation or late mobilisation post-surgery. One study included elderly patients (>65 years) undergoing elective spinal surgery for the correction of adult degenerative scoliosis [38], one study included patients undergoing lumbar discectomy [41] and one study included patients who had undergone anterior cervical discectomy and fusion, lumbar laminectomy, cervical nonfusion, and posterior laminectomy/foraminotomy [28]. Furthermore, one study included spine surgery for degenerative disease with low back pain and radiating pain (no more than two levels; 80% primary non-instrumented spondylodesis (50% in combination with decompression using autologous bone graft for posterolateral fusion), a small number of patients who underwent instrumented fusion with the same technique and a small number of patients underwent secondary surgery because of pseudarthrosis) [39]. One case of disc prosthesis at L5-S1 was also included in each group in this study.

### 3.3. Early Mobilisation Protocol

Mobilisation protocols varied between surgery type and between studies. Nielsen et al. combined an early rehabilitation programme with prehabilitation initiated two months prior to surgery and compared outcomes to patients receiving standard care [39]. Post-operatively, patients were intensively mobilised by a physiotherapist on the day of the operation and for 30 min, twice a day, in the days following surgery. In the control group, patients were only mobilised once a day. Exercises with a focus on improved muscle strength for the back and abdomen and cardiovascular conditioning were prescribed. The control group aimed for discharge on day eight, whereas the intervention group had an additional rehabilitation programme that aimed to discharge on the fifth postoperative day. Milestones for patients post-surgery were: Assisted positional change in bed, independent positional change in bed, assisted mobilisation to bedside, independent mobilisation to bedside, assisted mobilisation in walking frame, independent mobilisation to walking frame, independent personal hygiene, independent daily function on ward, walking without aids, complete training programme, independent stair climb and discharge to home [39].

In the study by Rupich et al. [28], a nurse-driven protocol was created to promote early mobility that would be easy to implement with uncomplicated neurosurgical spine patients. To establish early mobility goals and manage expectations, patient education began in the preadmission phase. Mobility was encouraged within six hours of arrival in the neuroscience unit from the post-anaesthesia care unit (PACU) to allow time for nurses to assess patients, treat pain and nausea as required and reiterate the goal of early mobility. Nurses and patient care technicians helped patients to get out of bed and ambulate without having to consult a physical therapist first. The goals for the day of surgery were to ambulate to the bathroom and to sit in a chair for meals and for postoperative day one, included being out of bed for all meals and ambulating in the hallway. On the day after surgery, nurses discontinued intravenous fluids and patient-controlled analgesia pumps at 6:00 am to help standardise care and set clear expectations. An early mobility algorithm was created to guide nurses, which separated posterior cervical fusion and lumbar fusion (may mobilise on postoperative day 0 depending on patient/clinical judgement) to cervical laminectomies and foramintomies, anterior cervical discectomy and lumbar laminectomies, foramintomies and discectomies. Exclusion criteria (cerebrospinal fluid leak with head of bed flat, hemodynamically unstable, uncontrolled pain) and a list of considerations (for example, patients refusing to get out of bed) were also included in the algorithm [28].

Qvarfordh et al. [41] conducted a pilot randomised controlled trial to investigate whether it was feasible and safe to mobilise patients shortly after lumbar disc surgery by allowing them to walk back from the PACU to the general ward. Patients in the intervention group were mobilised to sit, stand and visit the toilet at least one hour after surgery in the PACU. The patients rested in bed before they walked the 50 m to the general ward. They used a walking frame and were accompanied by a porter and a nurse with a wheelchair in case the patient should feel uncomfortable. In the control group, patients were not mobilised in the PACU, including visiting the toilet, and they were driven to the general ward whilst accompanied by a nurse. Once in the general ward, both groups were mobilised with assistance of a nurse or physiotherapist [41].

Adogwa et al. [38] did not describe their mobility protocol in detail but instead dichotomized patients into one of two groups based on the number of days from surgery to ambulation. Patients in the top and bottom quartiles were categorised as early ambulators or late ambulators, respectively. Early ambulators were patients who were mobilised within 24 h following surgery and late ambulators were patients who were mobilised at a minimum of 48 h after surgery [38].

### 3.4. Outcome Measures

#### 3.4.1. Postoperative Complications

Adogwa et al. [38] found the incidence of pneumonia to be significantly lower for early ambulators when compared to late ambulators (early ambulators: 1.51%, late ambulators: 8.47%, *p* = 0.04). Incidence of ilieus (early ambulators: 10.60%, late ambulators: 11.86%, *p* = 0.82), urinary tract infection (early ambulators: 6.06%, late ambulators: 8.47%, *p* = 0.64), deep vein thrombosis (early ambulators: 0%, late ambulators: 1.69%, *p* = 0.32), pulmonary embolism (early ambulators: 0%, late ambulators: 3.38%, *p* = 0.15), hematoma (early ambulators: 0%, late ambulators: 1.69%, *p* = 0.32), sensorimotor deficits (early ambulators: 1.51%, late ambulators: 1.69%, *p* = 0.93) and myocardial infarction (early ambulators: 0%, late ambulators: 3.38%, *p* = 0.15) were higher in the late ambulators, but these differences were non-significant. Incidence of urinary retention (early ambulators: 22.72%, late ambulators: 16.94%, *p* = 0.42) and delirium (early ambulators: 18.18%, late ambulators: 16.94%, *p* = 0.85) were higher in the early ambulation cohort but were also non-significant. Overall, late ambulators were 23.93% more likely to have at least one complication when compared to the early ambulators (early ambulators: 30.30%, late ambulators: 54.23%, *p* = 0.06).

Nielsen et al. [39] report no difference in postoperative complications between groups, with eight participants from each cohort having a major complication that required intensive treatment or secondary surgery (intervention group: severe pain (6); haematoma (1); allergic reaction (1); control group: severe pain (7); and haematomas (2)). Four patients in each group were reported to have minor complications (intervention group: urinary tract infection (2); and constipation (2); control group: wound infection (1); urinary retention (1); and constipation (2)).

Qvarfordh et al. [41] report levels of nausea as low in both their study groups and mobilisation did not worsen the level and incidence of nausea in either group. No vomiting was recorded, and only a few mild dizziness cases were registered in each group. Incidence of complication was recorded at three weeks follow up. In the intervention group, four patients had tenderness and swelling in the legs, and in the control group, there was one patient with complications. One patient from the intervention group had further surgery after eight days because of recurrent prolapse [41].

#### 3.4.2. Length of Stay and Discharge Disposition

Three studies report improved length of stay following an early mobilisation intervention post-spine surgery [28,38,39]. Nielsen et al. [39] found the intervention group left hospital significantly earlier than the control group (intervention group: median 5 (309) days, control group: 7 (5–15) days (*p* = 0.007)). Similarly, Adogwa et al. [38] report a 35% reduction in length of stay for early ambulators compared to late ambulators (early ambulators: mean 5.33 ± 3.02 days, late ambulators: 8.11 ± 7.70 days (*p* = 0.01)). In addition, the majority of patients in the early ambulation cohort were discharged directly home (71.21%), whereas only 22.03% of late ambulators were discharged straight home (*p* = 0.01). Discharge to a skilled nursing facility was higher in the late ambulation group (early ambulators: 28.78%, late ambulators: 62.71% (*p* = 0.001)), and 13.55% of late ambulators were discharged to an acute rehabilitation facility, compared to none of the early ambulation cohort (*p* = 0.00).

Rupich et al. [28] report a 6.7 h decrease in length of stay for lumbar laminectomy patients receiving an early mobility protocol compared to the control group (range: −12–(−1.5) (*p* = 0.012)). When adjustments had been made for age, sex, diabetes and number of vertebrae involved (two or more versus one), length of stay was decreased by 9.1 h (range: −13.9–(−4.3) (*p* < 0.001)). However, there were no significant differences in length of stay between groups for the anterior cervical discectomy and fusion, cervical nonfusion, and posterior laminectomy/foraminotomy procedure types both before and after adjusting for covariates. Qvarfordh et al. [41] found that patients in the intervention group stayed an average of 27 h in hospital (range: 21–32 h) whereas the control group spent 25 h (range: 21–49 h) in hospital.

#### 3.4.3. Readmission Rate

There were no differences in unplanned 30-day readmission rates reported in the study by Adogwa et al. [38] (early ambulators: 10.60%, late ambulators: 8.47%, (*p* = 0.55)).

#### 3.4.4. Performance-Based Functional Tests

Adogwa et al. [38] report the number of postoperative days between surgery and first ambulation to be significantly shorter for early ambulators compared to late ambulators (mean ± standard deviation (SD): 0.93 ± 0.24 days versus 2.83 ± 1.47 days (*p* = 0.01)). Likewise, the distance walked at first ambulation was almost two-fold greater for the early ambulation cohort (early ambulators: 116.34 ± 133.27 ft, late ambulators: 75.84 ± 94.14 ft (*p* = 0.05)), and significantly greater at discharge (early ambulators: 245.23 ± 270.75 ft, late ambulators: 132.20 ± 164.83 ft (*p* = 0.01)).

Once in the general ward, Qvarfordh et al. [41] report time to first mobilisation to be an average of 35 min (range: 20–270) for the intervention group and 180 min (range: 10–245) for the control group. During the first 24 h in the general ward, patients in the intervention group left their bed an average of nine times (range: 3–16) and patients in the control group an average of seven times (range: 4–11). Patients were out of bed for an average of 171 min (range: 50–670) and 210 (range: 90–420) min, respectively.

There were no differences in sit-to-stand or timed up and go scores recorded after intervention between groups in the study by Nielsen et al. [50]. However, the intervention group reached clinical recovery milestones faster than the control group (1–6 days vs. 3–13 days (*p* = 0.001)).

#### 3.4.5. Patient-Reported Outcome Measures

Nielsen et al. [39,40] report no difference in health-related quality of life scores following an integrated programme of prehabilitation and early rehabilitation. Qvarfordh et al. [41] found that patients who walked between the post-anaesthesia care unit to the general ward had lower opioid consumption than those who were driven. However, there were no differences in pain as reported by the numeric rating scale between the two groups before and after mobilisation. Nine out of the 11 patients in the intervention group reported a positive experience of walking back to the general ward and two patients would have preferred to be driven to the ward. In the control group, five out of the 11 patients said they had wanted to walk back. Nielsen et al. [39] found that significantly more patients in the intervention group were satisfied with their overall treatment and outcome when compared with the control group (15 out of 28 vs. 7 out of 32 (*p* = 0.02)).

#### 3.4.6. Compliance

Rupich et al. [28] report compliance to early mobility protocol using a monitoring form tracked by nurse practitioners throughout their study. Monthly compliance was calculated by dividing the number of patients who engaged in early mobility each month by the total number of patients who participated in the protocol in the same period. Average protocol compliance was calculated to be 87.3% per month, with common barriers reported to be: nurse’s lack of familiarity with protocol, no formal early mobility order, indwelling urinary catheters left in place in the operating room, and uncontrolled postoperative pain.

### 3.5. Early Mobilisation in Multimodal Interventions

Nine studies that were retrospective analyses of pre/post intervention data following implementation of a multimodal spinal surgery pathway (named either: Enhanced Recovery after Surgery, enhanced recovery after spine surgery, evidence based clinical care pathway, multidisciplinary committee meeting or perioperative protocol) were also included in a separate analysis and are summarised in Table 3 Procedures included in these studies were: discectomy, decompression, fusion and realignment operations to the cervical, thoracic and lumbar spine [21], tubular microdiscectomy and mini-open decompression as well as minimally invasive anterior or posterior fusion [46], laminoplasty [47], spine surgery for metastatic tumours [43], single-level microdiscectomy [44], lumbar spine fusion [42,45], lumbar and cervical surgery (less than or equal to four levels) [48] and multilevel lumbar or thoracolumbar spinal fusion [24].

These studies report outcomes as a total, and do not segregate out any effects related to each specific component of the intervention. It is important to note, however, that a multimodal, multidisciplinary approach to the care of a patient, inclusive of a specific early mobilisation initiated no later than day one post-surgery, is reported to improve length of hospital stay [21,42,44,45,46,47,48], healthcare costs [21,44,45], complication rate [24,44,48], pain or pain control [43,44,47] and patient satisfaction [21]. Compliance to early mobility protocol was recorded as 91.22% in one study [47], with those with severe weakness of the lower limbs unable to walk on postoperative day one. Conversely, one study did not track early mobilisation compliance (specific steps) as the information was not consistently available [43] and Chakravarthy et al. [24] describe monitoring for compliance with protocol to be challenging.

#### Early Mobilisation Protocol

The early mobilisation protocol varied between studies, but in general followed the same principles that patients should be mobilised, as soon as is safe, following surgery. In one study, the “Bums off Bed” initiative required consultants to visit patients on the day of surgery to encourage out of bed activity, including standing or marching on the spot with an aim of ensuring the patient knew that mobilisation was safe [21]. In most cases, it was the nurse or physiotherapist who was responsible for mobilising patients. In the study by Chakravarhty et al. [24], all patients without contraindication were mobilised by the nursing staff within eight hours of arriving to the regular nursing floor. If the patient was unable to be mobilised by the nursing staff, or was considered at high risk, an order for physiotherapy was automatically generated. The intervention delivered by Shields et al. [45] required patients to sit on the edge of the bed on the night of surgery, have two sessions of physiotherapy on postoperative day one, and walk in the hall and climb stairs on postoperative day two. Both Staarjtes et al. [46] and Tarikci Kilic et al. [44] encouraged patients to sit out of the bed for mobilisation within two hours after surgery. Early mobilisation in the study by Li et al. [47] involved on-bed movement on the day of surgery as well as sitting and assisted walking on postoperative day one.

Right after surgery, patients in the intervention delivered by Bradywood et al. [42] were required to log roll with help, walk to the doorway and were fitted with a brace but it was not required for movement. Recovery after surgery involved sitting at the edge of the bed, standing at the bedside and sitting in a chair. Patients were considered ready to leave once they could walk with supervision for multiple times a day and could use their brace when walking. Implementation of ERAS at a large cancer centre included getting patients out of bed by postoperative day one, encouraging movement three times daily and moving from the bed to the chair, and the chair to the bed with minimum assistance [43]. By discharge, patients were required to ambulate for 50 ft with or without assistive devices or if non-ambulatory, (i.e., wheelchair bound), to move from the bed to the chair, and from chair to bed with minimum assistance. Lastly, Sivaganesan et al. [48] encouraged early and frequent ambulation, with patient mobilisation to begin on postoperative day one. Following durotomy, the perioperative protocol recommended immediate mobilisation if a water-tight primary closure has been achieved, 24-h bed rest only if the primary repair was poor, and that patients should only lay flat if symptoms arose.

## 4. Discussion

The impact of early mobilisation on perioperative complications and length of stay has been demonstrated in a number of medical and surgical subspecialties, but its specific role in spinal procedures has not yet been elucidated [27]. This review found that despite the growth and adoption of ERAS to spine surgery, the evidence base for early mobilisation remains limited across all procedures. Our results indicate that goal-directed early mobilisation, delivered using an evidence-based algorithm with a clear, procedure-specific inclusion and exclusion criteria, may reduce length of stay and complication rate. In addition, there is evidence to suggest improved performance-based and patient-reported outcome measures when compared to bed rest following elective spinal surgery. However, as only four studies [28,38,39,41], all heterogeneous in their methodology, were found, we cannot provide procedure-specific recommendations for optimal mobilisation prescription.

Recovery following spinal surgery continues to be refined through improved multidisciplinary care. The evidence for early mobilisation included within enhanced recovery or multimodal interventions is encouraging; however, it is not possible to segregate the effects on outcomes from these studies [21,24,42,43,44,45,46,47,48]. Consistent with other surgical specialties, the implementation of enhanced recovery protocols in spine surgery are associated with a shorter length of stay and accelerated return to function without an increase to complications. Whilst these benefits are observed across several procedures and patient cohorts, the current evidence surrounding the use of ERAS protocols in spine surgery is largely restricted to retrospective reviews. These protocols are often informed from those used in other procedures, and given the lack of spine-specific evidence for each individual ERAS component [48], early mobilisation was most likely incorporated within these protocols using transferable evidence from other procedures. This is justified and underpinned by a sound physiological justification for avoiding immobilisation post-surgery. However, procedure-specific evidence should always be the aim in order to ensure that ERAS protocols remain evidence-based practice rather than an uncertain science [51].

Given the range of spinal procedures included within this review, it is perhaps not surprising to see the variation in delivery time of mobilisation intervention. For discectomies and microdiscectomies, mobilisation was encouraged within one [41] or two [44,46] hours following surgery. Patients undergoing non-complex lumbar fusion (less than six levels) were mobilised “right after surgery” [42] and those undergoing multilevel fusion of the lumbar or thoracolumbar spine within eight hours [24]. Following cervical laminectomies and foraminotomies, anterior cervical discectomy and fusion and lumbar laminectomies, foraminotomies and discectomies, patients were mobilised within six hours following surgery [28]. In the same study, mobilisation for patients undergoing posterior cervical fusion and lumbar fusion was still advised on the day of surgery, but based on individual and clinical judgement [28]. Some studies did not specify a time point, but instead considered early mobilisation to be within 24 h [38], on the day of surgery [21,39], on the night of surgery [45] or on postoperative day one [43,47,48].

In addition to differences in start times, the term ‘mobilisation’ is diverse in its meaning within this review. Definitions include: sitting on the edge of the bed [45], sitting out of bed [44], on-bed movement as well as sitting and assisted movement [47], a log roll and walk to the doorway [42], movement from the hospital bed to chair, then chair back to the bed, at a minimum assist level [43], standing and marching on the spot [21], sitting, standing, visiting the toilet and walking to the general ward [41] and “light activity in the room” including bed to chair, bed to bathroom and out of bed to bathroom movements [28]. In some excluded studies, early mobilisation was included within the intervention protocol but was not described in sufficient detail to warrant inclusion within this review [22,52,53,54].

Whilst this review reveals a lack of evidence to determine which early mobilisation intervention is most effective, mobilisation on the day of surgery and ambulation from the first postoperative day is possible across a range of different spinal procedures. Early mobility should be defined in future studies as standing and walking, which are both functional and clearly definable. Future studies should also provide clear details on mobilisation protocol, distance walked, assistance required and compliance to mobilisation goals. Further information on time of return from theatre and time from return to theatre to first mobilisation is also required to progress the evidence for early mobilisation in this patient population.

## 5. Limitations

This review is limited to studies published in the English language and therefore may have omitted relevant articles published in a different language. In addition, as previously mentioned, the results from the multimodal intervention cannot be solely attributed to the early mobilisation protocol.

## 6. Conclusions

This review summarises the pertinent research across a variety of elective spinal surgeries and suggests that early mobilisation is beneficial in terms of length of stay, postoperative complications, performance-based functional tests and patient-reported outcome measures. Whilst this is not surprising, given the evidence from other surgical specialities and known physiological effects of mobilising patients soon after surgery, the benefits of early mobilisation have been demonstrated across a range of patient groups and spinal procedures. Future work should aim to establish consensus-based, best practice guidelines on the optimal type and timing of mobilisation for individual spinal procedures. Once these have been established, compliance and outcome data are needed to further understand what prescription of early mobilisation should be administered in clinical practice.

## Figures and Tables

**Table 1:** PICOS (Population, Intervention, Comparison, Outcome measures, Study design) criteria.

PICOS Item	Inclusion Criteria	Exclusion Criteria
Population	Adults (over 18 years old) receiving elective spinal surgery Revision spinal surgery Surgery for scoliosis, kyphosis or lordosis Lumbar, cervical, thoracic or sacrum surgery	Children (under 18 years) receiving elective spinal surgery Non-elective surgery Coccyx surgery Surgery for spinal cord injury
Intervention	Early mobilisation (with out of bed activities starting no later than postoperative day one) Enhanced recovery (also termed “rapid recovery” or “fast-track”) programme inclusive of an early mobilisation intervention	Enhanced recovery programme without mobilisation protocol described Physical therapy intervention initiated later than postoperative day one Ambulatory surgery Neural mobilisation
Comparison	No early mobilisation (standard care) Best rest Pre/post comparison of early mobilisation intervention implementation.	No control group or comparison data
Outcome measures	Postoperative complication rate Length of hospital stay Patient disposition Readmission rate Performance-based function Patient-reported outcome measures Intervention adherence	Cost (economic evaluations)
Study design	Randomised clinical trials Non-randomised clinical trials Retrospective analyses Pilot randomised trials	Case studies Review articles Abstracts Editorials
Publication	Published in English Access to full text	Unpublished studies Study protocols

**Table 2:** Summary of studies (early mobilisation intervention only).

Study	Type of Surgery	Study Design	Patients	Early Mobilisation Protocol	Outcome Measures of Interest	Results
Adogwa et al. 2017 [38]	Elective spinal surgery (multilevel lumbar decompression and fusion for the correction of adult degenerative scoliosis)	Ambispective (early ambulation vs. late ambulation)	Early ambulators: *n* = 66, mean age 73.72 ± 6.25 years; Late ambulators: *n* = 59, mean age 73.22 ± 5.31 years	Early ambulators were mobilised within 24 h following surgery and late ambulators were at a minimum of 48 h after surgery	Postoperative complications, 30-day hospital readmission rate, postoperative functional status (duration to first ambulation, distance ambulated on the first day of ambulation, the distance of ambulation at discharge), LOS and discharge disposition.	There was a significantly lower incidence of pneumonia in the early ambulators (1.51% vs. 8.47%, *p* = 0.04). Late ambulators were more likely to have at least one complication (54.23% vs. 30.30%, *p* = 0.06). There were no significant differences for incidence of urinary retention, delirium, ileus, urinary tract infection, DVT, PE, hematoma, sensorimotor deficits or MI. The length of in hospital stay was 34% shorter in the early ambulators cohort (5.33 days vs. 8.11 days, *p* =0.01). Functional independence was superior in the early ambulators cohort, with the majority of patients discharged directly home after surgery compared with late ambulators (71.2% vs. 22.0%, *p* = 0.01)
Nielsen et al. 2008, 2010 [39,40]	Lumbar fusion or decompression for degenerative lumbar disease with low back pain and radiating pain	RCT (prehabilitation and early rehabilitation vs. standard care)	IG: *n* = 28, mean age 48 (range 31–80) years CG: *n* = 38, mean age 52 (38–88) years	The physiotherapist mobilised the patient of the day of the operation and 30 min twice daily in the following days. Exercises with a focus on improved muscle strength for the back and abdomen and cardiovascular conditioning were given. Milestones for patients post-surgery were: (1) Assisted positional change in bed; (2) Independent positional change in bed; (3) Assisted mobilisation to bedside; (4) Independent mobilisation to bedside; (5) Assisted mobilisation in walking frame; (6) Independent mobilisation to walking frame; (7) Independent personal hygiene; (8) Independent daily function on ward; (9) Walking without aids; (10) Complete training programme; (11) Independent stair climb; and (12) Discharge to home.	HRQOL, pain (BPI), function (Roland Morris Questionnaire, sit to stand, TUG, milestone achieved under hospitalisation), postoperative complications, and LOS.	At operation, the IG had improved function and postoperatively reached recovery milestones faster than the CG (1–6 days versus 3–13 days *p* = 0.001). LOS was shorter for the IG (5 days (range: 3–9) vs. 7 days (range: 5–15)) and no differences were recorded in postoperative complications, or adverse events. No difference in health-related quality of life scores was observed
Rupich et al. 2018 [28]	Anterior cervical discectomy and fusion, lumbar laminectomy, cervical nonfusion, and posterior laminectomy/foraminoteomy	Retrospective analysis (pre/post early mobility protocol implementation)	IG: *n* = 440, CG: *n* = 275	Cervical laminectomies and foraminotomies, anterior cervical discectomy and fusion and lumbar laminectomies, foraminotomies and discectomies: PO day 1: mobilise within 6 h of admission to floor, light activity in the room, bed to chair/bed to bathroom, out of bed to bathroom, no urinal/commode, out of bed for dinner. PO day 2: PCA/IVF discontinued at 6:00 am, foley removed at 6:00 am for lumbar fusions, increase mobility as tolerated, out of bed to bathroom, no urinal/commode, out of bed to chair for all meals starting with breakfast, may mobilise with patient care technician, do not have to wait for physiotherapist. Posterior cervical fusion and lumbar fusion: PO day 0: may mobilise based on individual/clinical judgement, PO day 1 as above	LOS, intervention adherence	Average compliance to protocol rates was 87.3% per month. Fall rates were monitored quarterly, and over one year the unit’s fall rates remained stable. Over a one-year period, implementation of the protocol resulted in a nine-hour reduction in LOS per hospitalisation in neurosurgical spine patients who underwent lumbar laminectomies. The protocol also allowed nurses more autonomy in patient care and was a catalyst for patient involvement in their postoperative mobility. There were no other statistically significant changes in LOS. The overall adjusted LOS reduction of 2.9 h for the IG was non-significant.
Qvarfordh et al. 2014 [41]	Lumbar discectomy	Pilot RCT	IG: *n* = 12, mean age 49 (28–60) years; CG: *n* = 11 mean age 44 (33–76) years	In the PACU, patients were mobilised to sit, stand and visit the toilet at least one hour after surgery. The patients rested in bed before they walked the 50 m to the general ward. They used a walking frame and were accompanied by a porter and a nurse with a wheelchair in case the patient feltuncomfortable. In the CG, patients were not mobilised in the PACU. They were driven to the general ward. Once in the general ward, both groups (IG and CG) were mobilised with assistance of a nurse or physiotherapist	Postoperative complications, pain, patient experience, time to mobilisation in the general ward and LOS	During the first 24 h postoperatively, IG had lower consumption of opioid compared with the CG, although there were no differences in pain between groups before and after mobilisation. In both groups, the level of nausea was low, and mobilisation did not worsen the level and incidence of nausea in either group. No vomiting was recorded, and only a few mild dizziness cases were registered in each group. Time to first mobilisation in the IG was 35 min (20–270) min and 180 (10–245) min in the CG. During the first 24 h in the general ward, patients in the IG left their bed an average of 9 times (3–16) and in the CG 7 times (4–11) and were out of bed for 171 min (50–670) and 210 (90–420) min, respectively. Patients in the IG were hospitalised for an average of 27 (21–32) h and in the CG for 25 (21–49) h.

DVT—deep vein thrombosis; PE—pulmonary embolism; MI—myocardial infarction; RCT—randomised controlled trial; IG—intervention group; CG—control group; BPI—brief pain inventory; TUG—timed up and go; HRQOL—health-related quality of life; IVF—intravenous fluid; PCA—patient-controlled analgesia; PO—postoperative; LOS—length of stay; PACU—post-anaesthesia care unit.

**Table 3:** Summary of studies (multimodal interventions).

Study	Type of Surgery	Study Design	Patients	Early Mobilisation Protocol	Outcome Measures of Interest	Results
Blackburn et al. 2016 [21]	Elective spinal surgery (discectomy, decompression, fusion and realignment operations to the cervical, thoracic and lumbar spine)	Retrospective analysis (pre/post ERAS implementation)	This study does not report the number of patients included in the analysis. The mean age of all patients at the institute is 55 years	The “Bums off Beds” initiative implemented as part of a Spinal Enhanced Recovery after Surgery programme in Musgrove Park Hospital, Taunton, recommended consultants to visit all patients postoperatively on the day of surgery to get them out of bed, either standing or marching on the spot to show them that mobilisation was safe	LOS, patient satisfaction, readmission rate	The pathway improved reliability of care, patient satisfaction and LOS (5.7 days at the start of the intervention to 2.7 days). A total of 100% of patients rated their care as “good” or “excellent”. The 52% reduction in length of stay was calculated to save the trust a theoretical £7800 per year (bed cost £174 per day)
Bradywood et al. 2017 [42]	Non-complex lumbar fusion (less than six levels)	Retrospective analysis (pre/post evidence based clinical care pathway implementation) adaption of “Lean”	244 (ERAS) vs. 214 (non-ERAS)	Right after surgery: log roll with help and walk to the doorway. Fitted for a corset brace but it is not required for movement. Recovery after surgery: sit on edge of the bed, stand at the bedside, and sit in a chair. Ready to leave: can walk with supervision multiple times each day, use brace when walking	LOS, patient disposition, pain and falls	LOS decreased from 3.9–3.4 days, a difference of 0.5 days (CI: 0.3, 0.8, *p* < 0.001). Discharge disposition also improved with 75% (183/244) of patients discharged to home post intervention versus 64% (136/214) pre intervention (*p* = 0.002). Patient satisfaction scores were not significantly changed
Chakravarthy et al. 2019 [24]	Multilevel lumbar or thoracolumbar spinal fusion	Retrospective analysis (pre/post ERAS implementation)	799 (ERAS) vs. 971 (non-ERAS)	All patients without contraindication were mobilised by the nursing staff within 8 h of arriving to the regular nursing floor. If the patient was unable to be mobilised by the nursing staff or was considered high risk, an order for physical therapy was automatically generated. Removal of the urinary catheter on POD 1 was encouraged to allow for easier mobilisation and to reduce the risk of urinary tract infection.	Infection prevention, blood management	Forty surgical site infections were seen in the pre-intervention cohort and 16 in the post-intervention group (4.12% vs. 2.00%; RR 0.48, 95% CI: 0.27–0.86; *p* = 0.01). Perioperative transfusion rates fell from 20.1% to 7.7% from pre intervention to post intervention (*p* = 0.004). There were no appreciable changes in morbidity or mortality rates
Grasu et al. 2018 [43]	Spine surgery for metastatic tumours	Retrospective analysis (pre/post ERSS implementation)	41 (ERSS) vs. 56 (non-ERSS)	Out of bed POD 1: movement 3 times daily, bed to chair, chair to bed at minimum assist level. By discharge: ambulate at minimum assist level for 50 ft with or without an assistive device; if non-ambulatory (i.e., wheelchair bound), bed to chair, chair to bed transfers at minimum assist level. Non-ERAS protocol = not consistently mobilised with frequency	Pain (NRS), LOS, readmission rate and postoperative complications	The ERSS group showed a trend toward better pain scores when compared to the with the pre-ERSS group. There were no significant differences in LOS, 30-day readmission rate, or 30-day complication rate observed between the two groups
Tarikci Kilic et al. 2019 [44]	Single-level lumbar microdiscetomy	Retrospective analysis (pre/post ERAS implementation)	60 (ERAS) vs. 60 (non-ERAS)	When the patients returned to the regular ward, they were encouraged to sit out of the bed for mobilisation within 2 h and oral intake was resumed as soon as possible	Time to first mobilisation, PONV, preoperative-postoperative VAS pain scores, postoperative analgesic requirement and LOS	LOS was shorter for the ERAS group (30.10 ± 7.80 h pre-ERAS and 26.52 ± 5.16 h ERAS). First oral intake and first mobilisation were earlier in the ERAS group. The incidence of PONV was less in the ERAS group. Postoperative analgesic requirements and postoperative VAS scores were significantly less in the ERAS group
Li et al. 2018 [47]	Laminoplasty	Retrospective analysis (pre/post ERAS programme implementation)	114 (ERAS) vs. 110 (non-ERAS)	Early mobilisation included on-bed movement on the day of surgery, as well as sitting and assisted walking on the postoperative day 1. Pre-ERAS, there was no requirement for postoperative mobilisation	Physiological function (early eating, mobilise), postoperative pain (VAS), LOS, postoperative complications, adverse reactions and protocol compliance	LOS was shorter for the ERAS group compared to the traditional care cohort (5.75 ± 2.46 vs. 7.67 ± 3.45 days, *p* < 0.001). The ERAS group ate earlier (8.45 ± 2.94 h vs. 21.64 ± 2.66 h, *p* < 0.001), mobilised earlier (assisted walking) (30.79 ± 14.45 vs. 65.24 ± 25.34 h, *p* < 0.001). Pain control was better in the ERAS group than traditional care group in terms of mean VAS score (2.72 ± 0.46 vs. 3.35 ± 0.46, *p* < 0.001) and mean maximum VAS score (3.76 ± 1.12 vs. 4.35 ± 1.15, *p* < 0.001) in 3 days after surgery. The morbidity rate was 21.05% (24 of 114 patients) in the ERAS group and 20.90% (23 of 110 patients) in the CG (*p* = 0.75). A total of 91.22% of ERAS patients performed bedside sitting and assisted walking on POD 1 (those with severe weakness of the lower limbs were unable to do so)
Shields et al. 2017 [45]	Spine fusion (except cervical) with and without major comorbidities	Retrospective analysis (2011–2014, multidisciplinary meetings initiated in 2011)	1978 (total)	Night of surgery: Patient sits on edge of bed. POD 1: Physical therapy on two occasions. POD 2: Walking in hall and climbing stairs.	LOS and readmission rate	LOS improved over the three years of intervention implementation. Average LOS for lumbar fusion (without major comorbidity) was statistically different for 2011–2012 vs. 2013–2014 (*p* < 0.001) and between 2012–2012 vs. 2011–2012 (*p* < 0.001)
Staartjes et al. 2019 [46]	Elective tubular microdiscectomy, mini-open decompression and minimally invasive anterior or posterior lumbar fusion	Retrospective analysis (ERAS implemented in 2013, data from 2013–2018 compared)	2595 (total)	Whenever feasible, early mobilisation was 2 h after operation under guidance of a physiotherapist	LOS, readmission rate, adverse events, reoperations, PROMs, function (ODI), health-related QOL (EQ-5D), pain (EQ-VAS), leg pain and back pain	Over the 5-year period, a trend toward a higher proportion of patients discharged home after a 1-night stay was observed (*p* < 0.001), with a concomitant decrease in adverse events in the overall cohort (*p* = 0.025) and without increase in readmissions. For fusion procedures, the rate of one-night hospital stays increased from 26% to 85% (*p* < 0.001). Likewise, the average LOS decreased steadily from 2.4 ± 1.2 days to 1.5 ± 0.3 days (*p* < 0.001). PROMS were vastly improved from baseline to 6 weeks, and an improvement in function was observed from week 6–1 year follow up. Health-related QOL and EQ-VAS also significantly improved, whilst neither leg nor back pain severities were further reduced
Sivaganesan et al. 2018 [48]	Elective lumbar or cervical surgery for the treatment of stenosis, disc herniation, spondylolisthesis, adjacent segment disease or pseudarthrosis	Retrospective analysis (pre/post implementation of perioperative protocol)	Preprotocol: *n* = 1596, Post protocol: *n* = 151	Bed rest after durotomy: Immediate mobilisation if water-tight primary closure achieved 24-h bed rest only if primary repair is poor. Patients should lay flat only if symptoms arise. Discharge planning to begin pre-surgery and patient mobilisation to begin on POD 1	90-day complication rate, EQ-5D, ODI, neck disability index, back and leg pain (NRS), patient satisfaction, LOS and discharge disposition	After protocol implementation, patients undergoing lumbar surgery had a significantly shorter LOS (2.5 ± 1.7 days versus 2.9 ± 2.2 days, *p* = 0.021) and lower complication rate (3.8% versus 12.8%, *p* = 0.002). No significant changes were noted for the PROMs, readmission rate, disability, pain and discharge disposition. For the cervical spine subgroup, there were no significant differences in any of the outcome measures. Overall, multivariate regression analyses demonstrated reduced length of stay (*p* = 0.013) and odds of 90-day complications (0.009) for post protocol patients

ERAS—Enhanced Recovery after Surgery; ERSS—Enhanced Recovery after Spine Surgery; POD—postoperative day; PROMS—patient-reported outcome measures; ODI—Oswestry Disability Index; QOL—quality of life; VAS—visual analogue scale; PONV—postoperative nausea and vomiting; NRS—numeric rating scale; LOS—length of stay; EQ-5D—EuroQol five-dimension scale; CI—confidence interval; CG—control group; RR—relative risk.

## Appendix A

**Table A1:** Modified Downs and Black Checklist, UTD—Unable to determine.

Item	Description	Nielsen et al. [39]	Rupich et al. [28]	Qvarfordh et al. [41]	Adogwa et al. [38]
1	Is the hypothesis/aim/objective of the study clearly described?	Yes (1)	Yes (1)	Yes (1)	Yes (1)
2	Are the main outcomes to be measured clearly described in the Introduction or Methods section?	Yes (1)	Yes (1)	Yes (1)	Yes (1)
3	Are the characteristics of the patients included in the study clearly described?	Yes (1)	Yes (1)	Yes (1)	Yes (1)
4	Are the interventions of interest clearly described?	Yes (1)	Yes (1)	Yes (1)	Yes (1)
6	Are the main findings of the study clearly described?	Yes (1)	Yes (1)	Yes (1)	Yes (1)
7	Does the study provide estimates of the random variability in the data for the main outcomes?	Yes (1)	Yes (1)	Yes (1)	Yes (1)
8	Have all important adverse events that may be a consequence of the intervention been reported?	Yes (1)	Yes (1)	Yes (1)	Yes (1)
10	Have actual probability values been reported?	Yes (1)	Yes (1)	No (0)	Yes (1)
**External validity**					
11	Were the subjects asked to participate in the study representative of the entire population from which they were recruited?	Yes (1)	Yes (1)	Yes (1)	UTD (0)
12	Were those subjects who were prepared to participate representative of the entire population from which they were recruited?	Yes (1)	Yes (1)	Yes (1)	UTD (0)
13	Were the staff, places, and facilities where the patients were treated, representative of the treatment the majority of patients receive?	Yes (1)	Yes (1)	Yes (1)	Yes (1)
**Internal validity**					
17	In trials and cohort studies, do the analyses adjust for different lengths of follow-up of patients, or in case-control studies, is the time period between the intervention and outcome the same for cases and controls?	Yes (1)	Yes (1)	Yes (1)	Yes (1)
18	Were the statistical tests used to assess the main outcomes appropriate?	Yes (1)	Yes (1)	UTD (0)	Yes (1)
19	Was compliance with the intervention/s reliable?	No (0)	No (0)	Yes (1)	Yes (1)
20	Were the main outcome measures used accurate (valid and reliable)?	Yes (1)	Yes (1)	Yes (1)	Yes (1)
21	Were the patients in different intervention groups (trials and cohort studies) or were the cases and controls (case-control studies) recruited from the same population?	Yes (1)	Yes (1)	Yes (1)	Yes (1)
22	Were study subjects in different intervention groups (trials and cohort studies) or were case and controls (case control studies) recruited over the same period of time?	Yes (1)	No (0)	Yes (1)	Yes (1)
**Total**		**16 (94%)**	**15 (88%)**	**15 (88%)**	**15 (88%)**
